# Identification of cuproptosis-related diagnostic biomarkers in idiopathic pulmonary fibrosis

**DOI:** 10.1097/MD.0000000000036801

**Published:** 2024-01-12

**Authors:** Qi Wang, Yu Shang, Yupeng Li, Xincheng Li, Xue Wang, Yaowu He, Jing Ma, Shangwei Ning, Hong Chen

**Affiliations:** a Department of Pulmonary and Critical Care Medicine, Second Affiliated Hospital of Harbin Medical University, Harbin, China; b Department of Respiration, The First Hospital of Harbin, Harbin, China; c College of Bioinformatics Science and Technology, Harbin Medical University, Harbin, China.

**Keywords:** biomarker, cuproptosis, idiopathic pulmonary fibrosis, immune infiltration

## Abstract

Idiopathic pulmonary fibrosis (IPF) is a progressive and fatal lung disease with clinical and pathological heterogeneity. Recent studies have identified cuproptosis as a novel cell death mechanism. However, the role of cuproptosis-related genes in the pathogenesis of IPF is still unclear. Two IPF datasets of the Gene Expression Omnibus database were studied. Mann–Whitney U test, correlation analysis, functional enrichment analyses, single-sample gene set enrichment analysis, CIBERSORT, unsupervised clustering, weighted gene co-expression network analysis, and receiver operating characteristic curve analysis were used to conduct our research. The dysregulated cuproptosis-related genes and immune responses were identified between IPF patients and controls. Two cuproptosis-related molecular clusters were established in IPF, the high immune score group (C1) and the low immune score group (C2). Significant heterogeneity in immunity between clusters was revealed by functional analyses results. The module genes with the strongest correlation to the 2 clusters were identified by weighted gene co-expression network analysis results. Seven hub genes were found using the Cytoscape software. Ultimately, 2 validated diagnostic biomarkers of IPF, CDKN2A and NEDD4, were obtained. Subsequently, the results were validated in GSE47460. Our investigation illustrates that CDKN2A and NEDD4 may be valid biomarkers that were useful for IPF diagnosis and copper-related clustering.

## 1. Introduction

Idiopathic pulmonary fibrosis (IPF), a chronic, progressive, and fibrotic interstitial lung disease, has a persistent decline in lung function and progressive worsening of symptoms.^[1,2]^ IPF patients have a poor prognosis, with a median survival of 3 to 5 years.^[3]^ Currently, the only approach to IPF is lung transplantation, and pirfenidone and nintedanib can only delay the progression of the disease.^[4]^ Therefore, there is an urgent need to explore valid new diagnostic biomarkers and therapeutic targets for IPF in order to save more patients’ lives.

Trace metals, such as iron and copper, are necessary to maintain the biological activity of cells.^[5,6]^ One of the mechanisms of cell death associated with metal ions, ferroptosis, has been studied in various diseases, such as IPF, lung adenocarcinoma, and liver cancer.^[7–9]^ A recent remarkable discovery revealed that increased intracellular copper concentrations could induce lipid acylation of the tricarboxylic acid cycle proteins, leading to a novel type of cell death known as cuproptosis.^[6]^ Copper is an essential component of many biological processes, and its metabolic disorders play an important role in angiogenesis and metastasis.^[10,11]^ It has been shown that IPF patients have an imbalance of angiogenic regulators and that elevated vascular endothelial growth factor (VEGF), a key regulator of angiogenesis, is considered an important marker of severity.^[12]^ However, the relationship between cuproptosis-related genes (CRGs) and the pathogenesis of IPF is unclear.

In the present study, we systematically studied for the first time the analysis of cuproptosis-related differential expressed genes (DEGs) and immune infiltration between IPF patients and normal controls. IPF patients were classified into 2 clusters based on cuproptosis-related DEGs and functional analyses were performed. Subsequently, weighted gene co-expression network analysis (WGCNA) was used to identify the most significant module genes in relation to IPF and normal controls. Receiver operating characteristic (ROC) curve analysis was performed on cuproptosis-related DEGs and hub genes in the module to determine the diagnostic value. Finally, GSE47460 (GPL6480) and GSE47460 (GPL14550) were used for verification. In conclusion, the present study is the first to link IPF to cuproptosis in search of valid diagnostic biomarkers. An innovative approach was used to find new effective biomarkers of IPF.

## 2. Materials and methods

### 2.1. Acquisition and preprocessing of datasets

Two datasets (GSE32537 and GSE47460) about IPF were obtained from the Gene Expression Omnibus database (http://www.ncbi.nlm.nih.gov/geo/). More detailed information was available in Table S1 (Supplemental Digital Content, http://links.lww.com/MD/L222). Percent predicted forced vital capacity (FVC% predicted), percent predicted lung diffusion capacity for carbon monoxide (DLCO% predicted), and St George Respiratory Questionnaire were obtained from GSE32537. Ten CRGs [dihydrolipoamide dehydrogenase (DLD), dihydrolipoamide S-acetyltransferase (DLAT), ferredoxin 1 (FDX1), lipoic acid synthetase (LIAS), lipoyltransferase 1 (LIPT1), pyruvate dehydrogenase E1 subunit alpha 1 (PDHA1), pyruvate dehydrogenase E1 subunit beta (PDHB), cyclin dependent kinase inhibitor 2A (CDKN2A), glutaminase (GLS), and metal regulatory transcription factor 1 (MTF1)] were received from a previous study.^[6]^ Due to the availability of public information resources, ethics committee approval was not required.

### 2.2. Functional analysis

CIBERSORT is a deconvolution algorithm based on gene expression data, which has a composition of 22 infiltrating immune cells. These infiltrated immune cell compositions were structured as a leukocyte gene signature matrix designated LM22 (Table S2, Supplemental Digital Content, http://links.lww.com/MD/L223) obtained from a former publication.^[13]^ Single sample gene set enrichment analysis (ssGSEA) was performed to calculate the infiltration score of 19 immune cells and the activity of 15 immune-related pathways based on the “GSVA” R package (v.1.40.1).^[14]^ These genes associated with immune cells^[15]^ are shown in Table S3 (Supplemental Digital Content, http://links.lww.com/MD/L224). The Wilcoxon test was used to test whether the difference between the 2 groups studied was statistically significant. Gene Ontology (GO) and Kyoto Encyclopedia of Genes and Genomes (KEGG) analysis were carried out using the “clusterProfiler” R package (v.4.4.4).^[16]^ *P* values were adjusted with the Benjamini & Hochberg (BH) method. Gene set enrichment analysis^[17]^ can be performed to analyze gene expression data and obtain enriched biological pathways in a phenotypically related genomic distribution using the “clusterProfiler” R package. A permutation test with 1000 times was run to determine important pathways (*P* value < .05). Immune score, stromal score and ESTIMATE score were calculated based on the “estimate” R package (v.1.0.13).^[18]^

### 2.3. Unsupervised clustering of IPF patients

Based on the expression profiles of the 5 CRGs (DLAT, CDKN2A, DLD, GLS, and LIAS), we applied unsupervised clustering analysis in the Sangerbox tools, a free online platform for data analysis (http://sangerbox.com/).^[19]^ We set the following parameters: the clustering method was pam, the clustering distance was based on spearman, the maximum number was 6, the sampling number was 10, and the sampling ratio was 0.8. Based on the expression of the above 5 genes in the 2 clusters, principal component analysis (PCA) was performed using the PCA analysis visualization tool function in the SangerBox platform.

### 2.4. Weighted gene co-expression network analysis (WGCNA)

WGCNA, a common method for analyzing the relationship between disease phenotypes and genes, was performed in order to identify co-expression modules using the “WGCNA” R package (v.1.71).^[20]^ We constructed the scale-free network by calculating various power values. A scale-free R2 of 0.9 was the appropriate power value we chose. Cluster analysis was used to identify highly similar modules and a minimum module size set to 30. The TOM dissimilarity (1-TOM) based on hierarchical clustering tree algorithm was used to obtain different color modules. Module significance (MS) and gene significance (GS) represent: the relationship between module and disease status, and the correlation between gene and clinical phenotype, respectively.

### 2.5. Protein–protein interaction network and hub genes

Protein–protein interaction (PPI) network was constructed and visualized with Cytoscape software (version 3.7.2).^[21]^ The node file and eged file generated by WGCNA were used for PPI analysis and hub genes identification using Cytohubba plugins in Cytoscape software. The colors of the gene nodes referred to various degree values.

### 2.6. Evaluation of diagnostic efficiency

To investigate whether genes would have diagnostic efficiency in distinguishing IPF from controls, we carried out ROC curve analysis in SPSS (version 25.0). The combined diagnostic value of the 2 genes was judged based on the predicted probability of binary logistic regression. Diagnostic ability was assessed according to the area under the curve (AUC) values.

### 2.7. Statistical analysis

Mann–Whitney U test was applied to have a comparison of gene expression levels in each group. With the help of the “corrplot” R package (https://CRAN.R-project.org/package=corrplot, v.0.92), we performed correlation analysis and plotted correlation graphs. Statistical analyses and images creation were executed by GraphPad Prism (version 9.0), Cytoscape software (version 3.7.2), SPSS (version 25.0), and R software (version 4.1.0). A two-tailed *P* value < .05 was considered statistically significant. P.adjust was obtained by the BH method. Heatmaps were plotted by means of the “pheatmap” R package (https://CRAN.R-project.org/package=pheatmap, v.1.0.12).

## 3. Results

**Figure 1.**
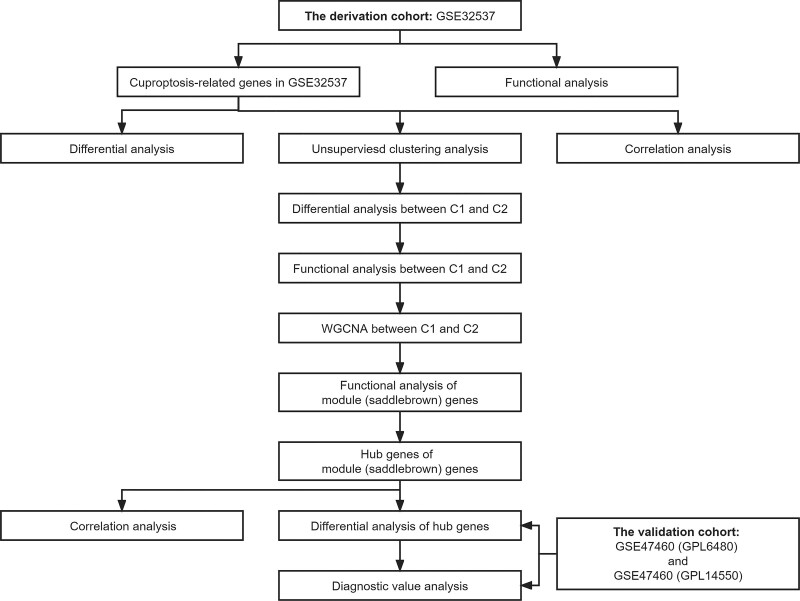
Flow chart of study design.

The workflow diagram for this study was shown in Figure 1 The baseline table containing characteristics of all datasets were presented in Table S1 (Supplemental Digital Content, http://links.lww.com/MD/L222).

### 3.1. Abnormalities of CRGs and immune infiltration in IPF patients

**Figure 2.**
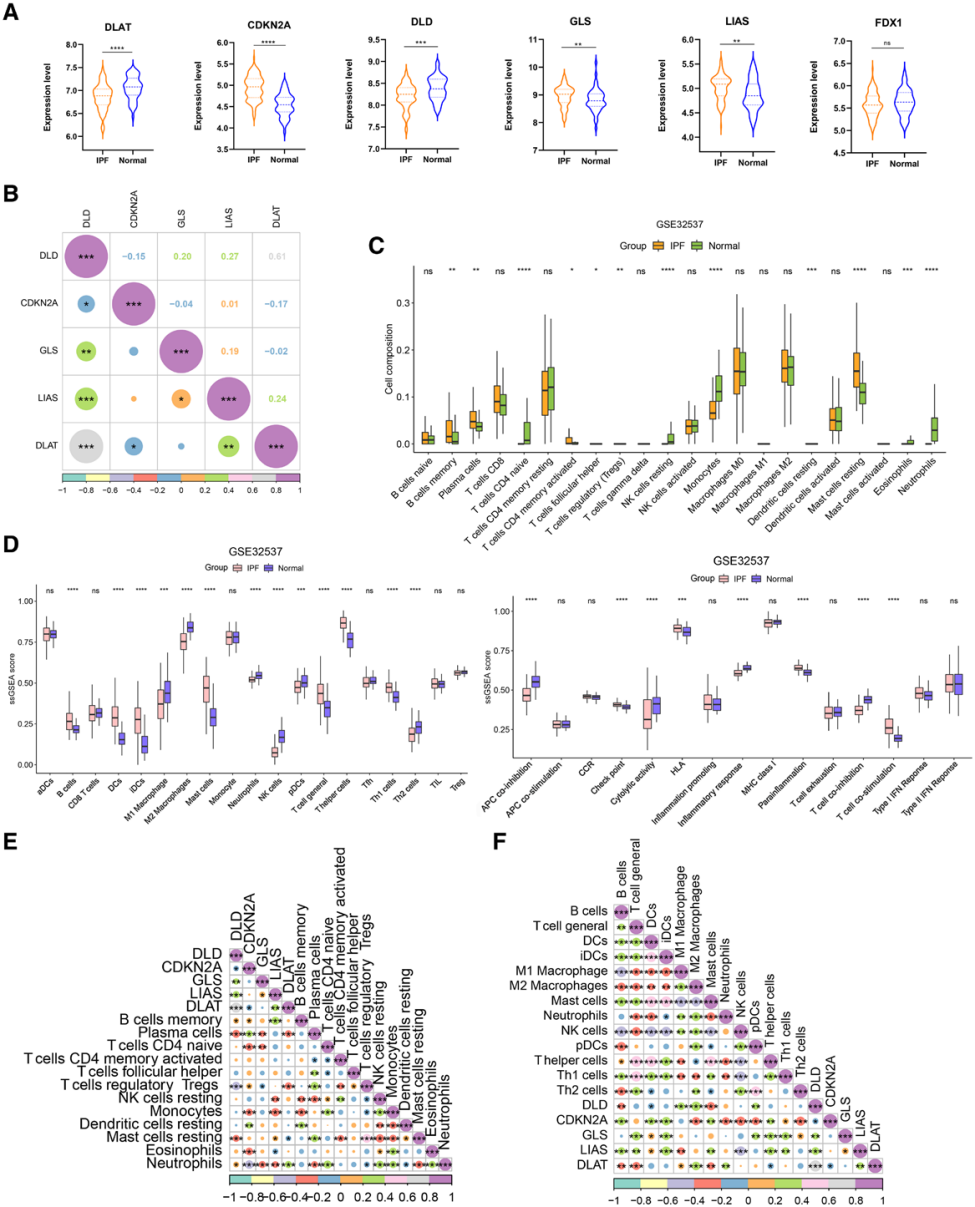
Identification of cuproptosis-related DEGs between IPF and normal controls in GSE32537. (A) Expression levels of 6 cuproptosis-related DEGs. (B) Correlation analysis between 5 cuproptosis-related DEGs in IPF patients and normal controls. (C) CIBERSORT result between IPF and normal controls. (D) ssGSEA result between IPF and normal controls. (E) Correlation analysis between 5 cuproptosis-related DEGs and different immune infiltrating cells between IPF and control from CIBERSORT result. (F) Correlation analysis between 5 cuproptosis-related DEGs and different immune infiltrating cells between IPF and control from ssGSEA result. *P < .05, **P < .01, ***P < .001, ****P < .0001.

To elucidate the role of CRGs in the development and progression of IPF, we first assessed the expression levels of 10 CRGs in the GSE32537 between IPF and controls. In this dataset, only 6 out of 10 genes were present, and 5 of them were DEGs. CDKN2A, GLS, and LIAS were upregulated in IPF. However, DLAT and DLD were down-regulated DEGs (Fig. 2A). The results of correlation analysis of 5 DEGs showed that DLD was significantly correlated with other genes (Fig. 2B). The highest correlation coefficient was 0.61 between DLD and DLAT. There were significant differences in immune infiltration between IPF and control groups as indicated by the ssGSEA and CIBERSORT results. CIBERSORT results showed high levels of B cells memory, plasma cells, T cells CD4 memory activated, mast cells resting in IPF patients. There were lower levels of T cells CD4 naive, NK cells resting, monocytes, eosinophils, and neutrophils in IPF patients (Fig. 2C). The ssGSEA results revealed that B cells, dendritic cells, iDCs, mast cells, T cell general, T helper cells, Th1 cells, check point, HLA, parainflammation, and T cell co-stimulation levels were higher in IPF patients. Lower levels of neutrophils, NK cells, pDCs, Th2 cells, adenomatous polyposis coli (APC) co-inhibition, cytolytic activity, inflammatory response, and T cell co-inhibition was observed in IPF patients (Fig. 2D). To investigate whether there was significant correlation between cuproptosis-related DEGs and immune cells, we performed a correlation analysis. A significant correlation between cuproptosis-related DEGs and many immune cells was found from the results (Fig. 2E and F). The results demonstrated significant correlation between CDKN2A and more immune cells, positive correlation with B cells and DCs, and negative correlation with neutrophils, monocytes and M2 macrophages. These results implied that cuproptosis-related DEGs might be a key factor in regulating the immune infiltration status of IPF patients.

### 3.2. Cuproptosis clusters in IPF patients

**Figure 3.**
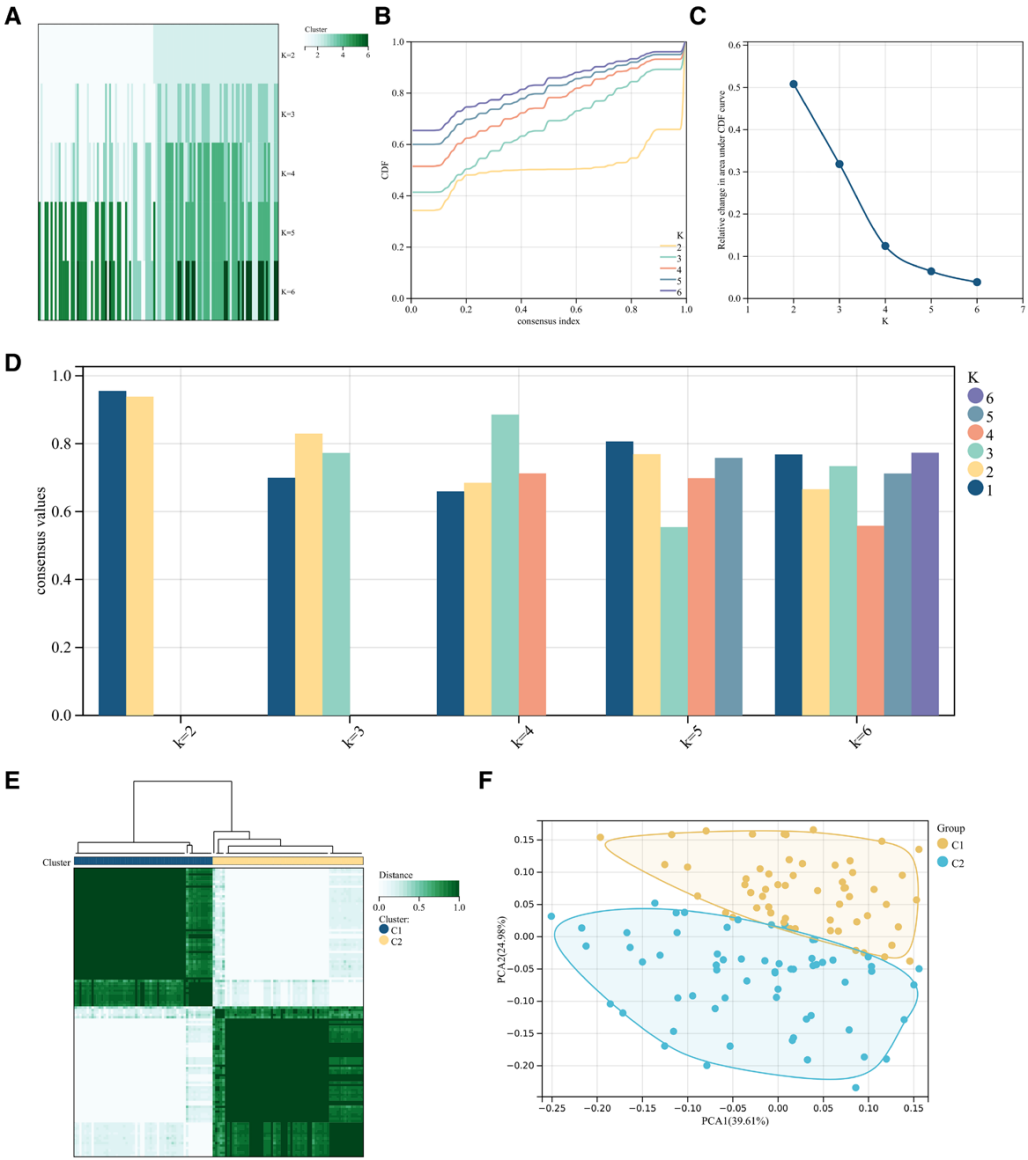
Identification of cuproptosis clusters in IPF patients. (A) The classification when k = 2–6. (B) CDF curves. (C) CDF delta area curves. (D) Values of consensus clustering. (E) The consensus matrixes for IPF patients based on the DEGs between 2 cuproptosis-related clusters. (F) PCA plot of 2 cuproptosis-related clusters. CDF = Cumulative distribution function.

In order to analyze IPF patients according to CRGs, unsupervised clustering analysis was carried out. Two cuproptosis-related phenotypes were identified, called cluster 1 (C1) and cluster 2 (C2) (Fig. 3A–F). Only when k = 2, the consensus values of each subtype was > 0.9 (Fig. 3D). Based on the results of PCA, a significant difference could be noted between C1 and C2 (Fig. 3F).

**Figure 4.**
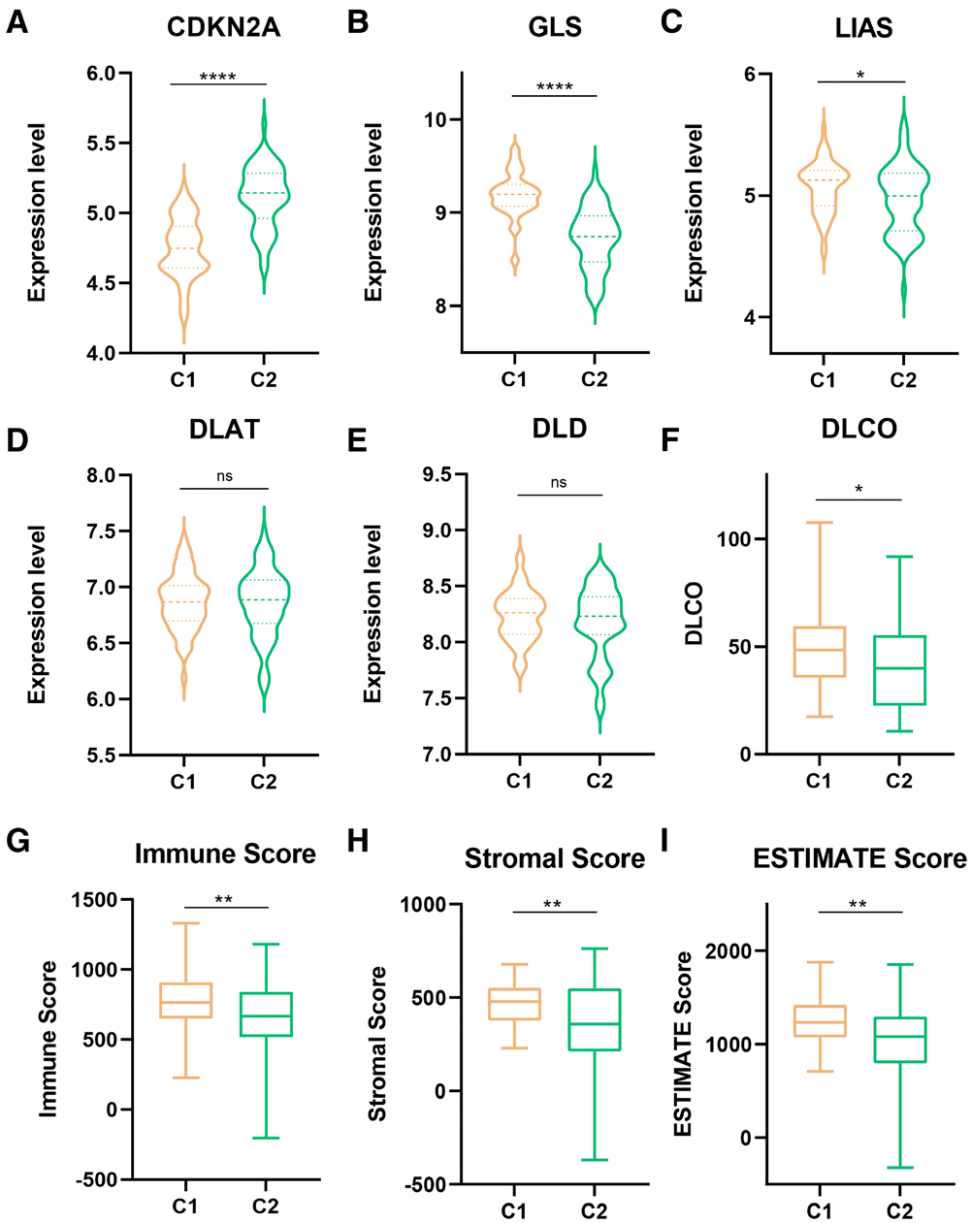
Levels of cuproptosis-related DEGs and characteristics between the 2 cuproptosis-related clusters. (A–E) Expression levels of CDKN2A, GLS, LIAS, DLAT, and DLD between C1 and C2. (F) DLCO% predicted between C1 and C2. (G-I) Immune score, stromal score, and ESTIMATE score between C1 and C2. *P < .05, **P < .01, ***P < .001, ****P < .0001.

To further investigate the differences between C1 and C2, we investigated the gene expression levels, pulmonary function, immune score, stromal score, ESTIMATE score, and smoking status of the 2 clusters. The differences in expression levels between C1 and C2 for 3 of the 5 DEGs were statistically significant (Fig. 4A–E). The expression levels of GLS and LIAS were lower and the expression level of CDKN2A was higher in C2 (Fig. 4A–C). Compared to C1, DLCO% predicted was lower in C2 (Fig. 4F). Meanwhile, immune score, stromal score, and ESTIMATE score were lower in C2 (Fig. 4G–I). Therefore, we called C1 as the high immune score group and C2 as the low immune score group. However, the differences in FVC% predicted, St George Respiratory Questionnaire score and smoking status between C1 and C2 were not statistically significant (Fig. S1A–C, Supplemental Digital Content, http://links.lww.com/MD/L218).

### 3.3. Functional analyses for cuproptosis clusters

**Figure 5.**
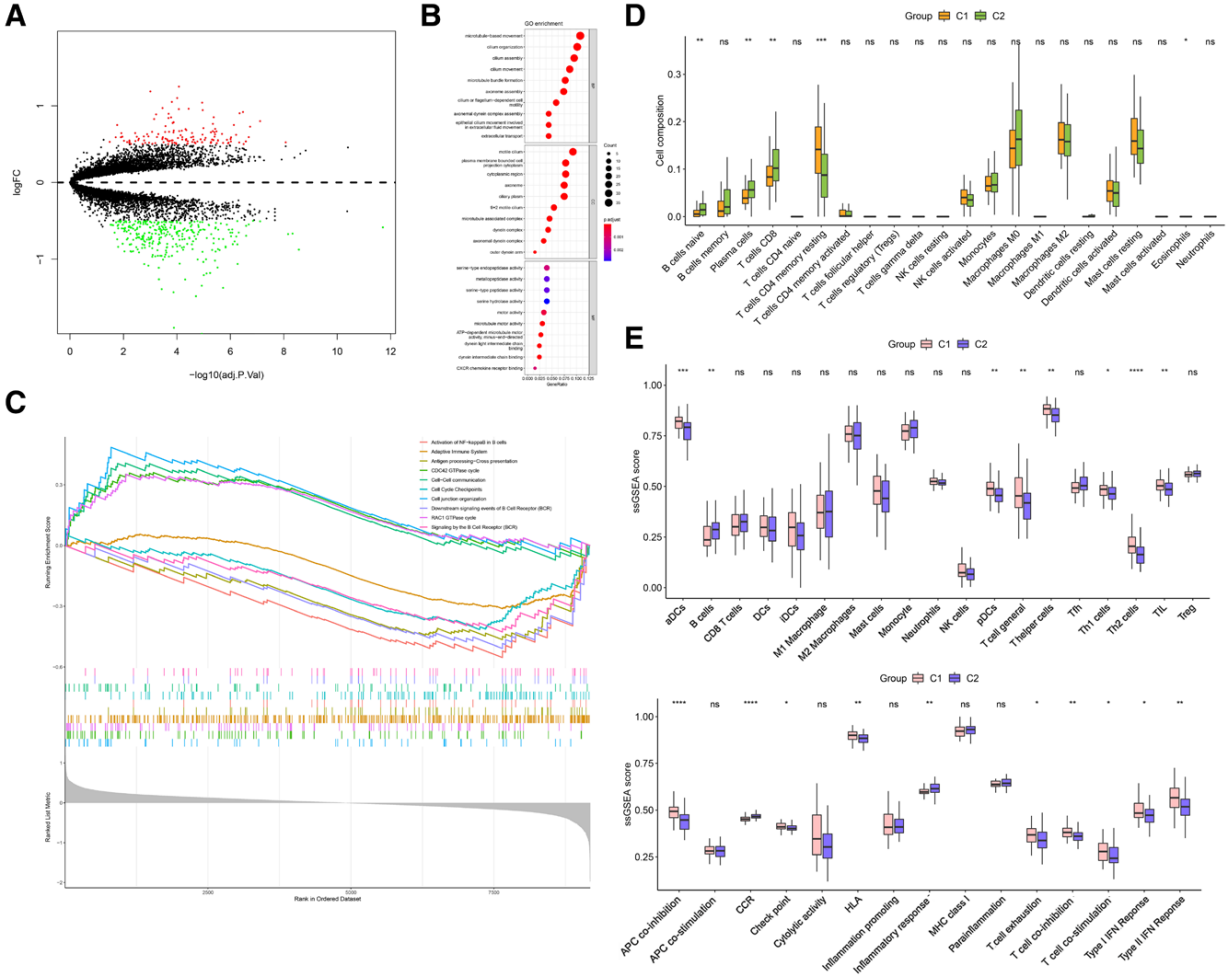
Functional enrichment analyses of the 2 cuproptosis-related clusters. (A) A volcano map of DEGs between C1 and C2. (B) GO enrichment analysis result. (C) GSEA result between C1 and C2. (D) CIBERSORT result between C1 and C2. (E) ssGSEA result between C1 and C2.

To further explore the functional mechanisms of C1 and C2 in IPF patients, we performed a series of functional enrichment analyses. A volcano plot demonstrated the distribution of DEGs between C1 and C2 (Fig. 5A). According to the result of GO analysis, these DEGs were closely related to cilium and cilia motility (Fig. 5B). The result of GSEA analysis showed that DEGs were significantly associated with the immune system, such as activation of NF-kappaB in B cells, adaptive immune system, downstream signaling events of B cell receptor (BCR) and signaling by the BCR (Fig. 5C). The above results were further elaborated based on the CIBERSORT and ssGSEA results. CIBERSORT results showed that the low immune score group contained lower T cells CD4 memory resting, higher B cells naive, plasma cells, and T cells CD8 (Fig. 5D). The ssGSEA results showed that ssGSEA scores of B cells, CCR, and inflammatory response were higher and ssGSEA scores of aDCs, pDCs, T cell general, T helper cells, Th1 cells, Th2 cells, TIL, APC co-inhibition, check point, HLA, T cell exhaustion, T cell co-inhibition, T cell co-stimulation, type I IFN response, and type II IFN response were lower in the low immune score group (Fig. 5E).

### 3.4. Weighted gene co-expression network analysis and module analysis

**Figure 6.**
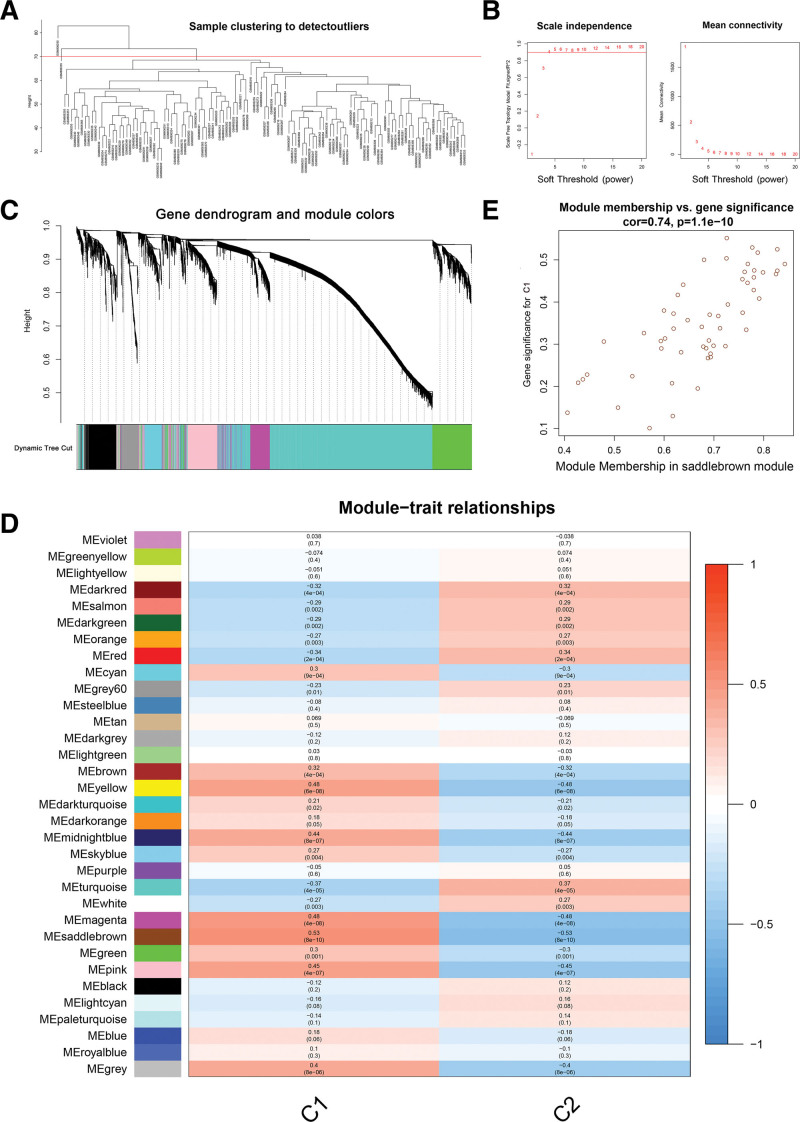
WGCNA between the 2 cuproptosis-related clusters. (A) Sample clustering to detect outliers. (B) The choice of soft thresholds power. (C) Cluster dendrogram of genes with the specified module color. (D) Module-trait relationships between C1 and C2. (E) A scatterplot between module membership and gene significance in the saddlebrown module containing 55 genes.

To identify key gene modules associated with C1 and C2, we established co-expression networks and modules for C1 and C2 patients based on the WGCNA algorithm. Outlier samples were removed by us with a height setting of 70 (Fig. 6A). Co-expressed gene modules were recognized with a scale-free R2 equal to 0.9 and a soft power value set to 4 (Fig. 6B). Gene clustering tree diagram displayed 33 co-expression modules in different colors (Fig. 6C). The heatmap of module-trait relationships was used to demonstrate the correlation analysis of modal characteristics with clusters (Fig. 6D). Correlation analysis revealed that the 55 saddlebrown module genes were significantly associated with both C1 and C2 (Fig. 6D and E).

### 3.5. Identification of hub genes and diagnostic biomarkers

**Figure 7.**
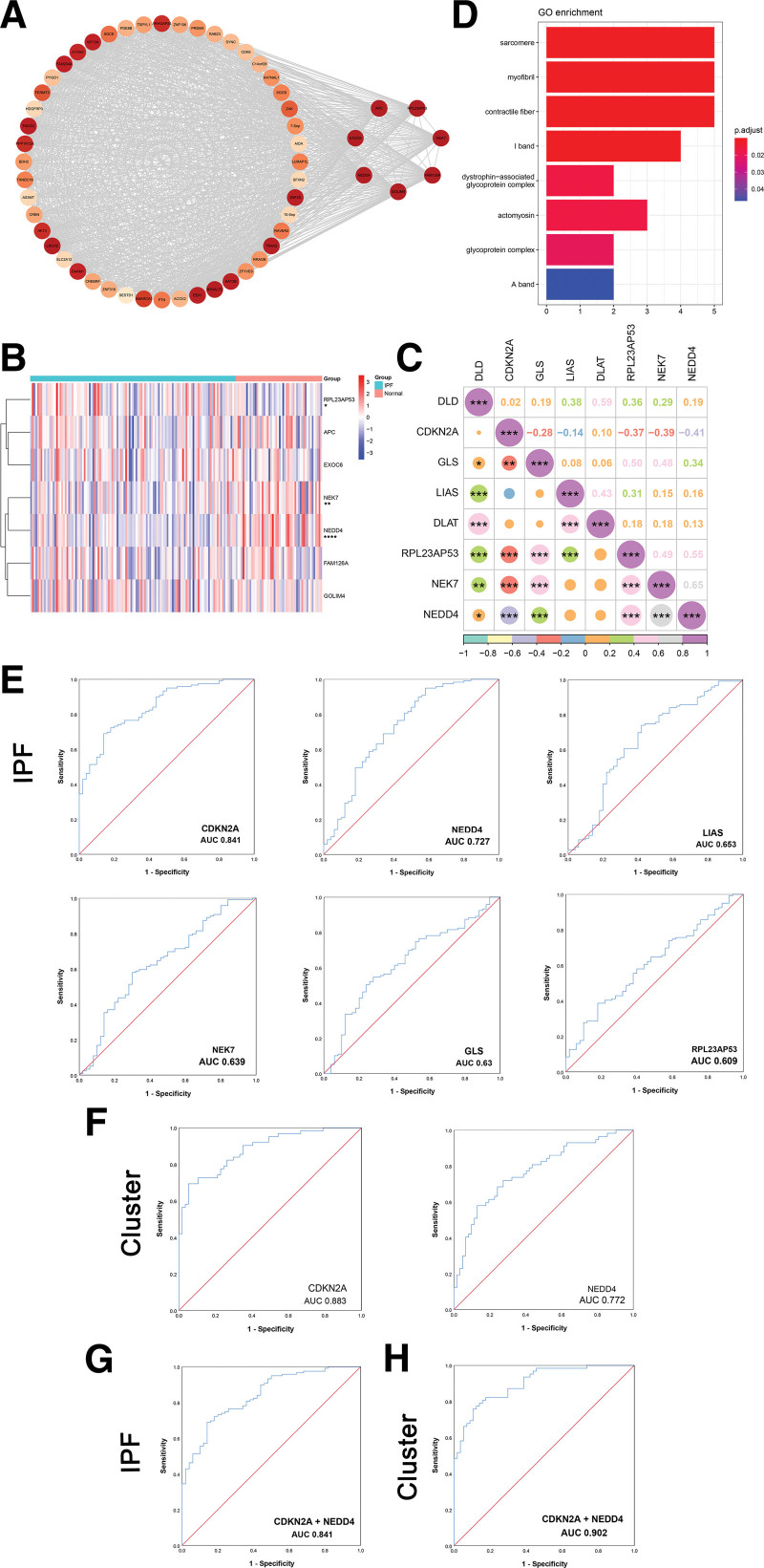
Identification of hub genes and diagnostic biomarkers. (A) Seven hub genes from the saddlebrown module. (B) Heatmap of 7 hub genes between IPF and normal controls. (C) Correlation analysis between 5 cuproptosis-related DEGs and significantly different hub genes in IPF patients. (D) GO enrichment analysis result of 55 saddlebrown module genes. (E) ROC analyses of CDKN2A, NEDD4, LIAS, NEK7, GLS, and RPL23AP53 for IPF. (F) ROC analyses of CDKN2A and NEDD4 for cuproptosis-related clusters. (G) ROC analysis result of combined diagnosis for IPF. (H) ROC analysis result of combined diagnosis for cuproptosis-related clusters. *P < .05, **P < .01, ***P < .001, ****P < .0001.

To further investigate the genes in saddlebrown module, a PPI network and hub genes were obtained by Cytoscape software based on the results of nodes and edges of WGCNA. Seven hub genes were obtained by us, APC protein, ribosomal protein L23a pseudogene 53 (RPL23AP53), NIMA related kinase 7 (NEK7), hyccin (FAM126A), golgi integral membrane protein 4 (GOLIM4), NEDD4 E3 ubiquitin protein ligase (NEDD4), and exocyst complex component 6 (EXOC6), respectively (Fig. 7A, Fig. S2A–G, Supplemental Digital Content, http://links.lww.com/MD/L219). Specifically, there were 3 DEGs, up-regulated RPL23AP53 and down-regulated NEDD4 and NEK7, between IPF and controls (Fig. 7B, Fig. S3A–G, Supplemental Digital Content, http://links.lww.com/MD/L220). By correlation analysis of the above 3 DEGs with cuproptosis-related DEGs, the result showed that NEDD4 and NEK7 are positively correlated with the largest correlation coefficient of 0.65 (Fig. 7C). It was worth mentioning that the GO enrichment analysis showed that 55 modular genes were strongly correlated with sarcomere and myofibril, etc (Fig. 7D). GSEA result showed that these genes were enriched in signal transduction (Fig. S4A, Supplemental Digital Content, http://links.lww.com/MD/L221).

To identify valid diagnostic biomarkers, ROC curves were performed. As shown in Figure 7E, the AUC values of CDKN2A, NEDD4, LIAS, NEK7, GLS, and RPL23AP53 at the time of IPF diagnosis were 0.841, 0.727, 0.653, 0.639, 0.63, and 0.609, respectively. The AUC values of CDKN2A and NEDD4 > 0.7, so CDKN2A and NEDD4 had excellent ability to diagnose IPF. In the differential diagnosis of C1 and C2, the AUC values of CDKN2A and NEDD4 were 0.883 and 0.772, respectively (Fig. 7F). The AUC value for the combined diagnosis of CDKN2A and NEDD4 were 0.841 (Fig. 7G). Surprisingly, the AUC value for the combined differential diagnosis of C1 and C2 was 0.902 (Fig. 7H). To explore whether there was any correlation between the 3 genes and lung function, we performed correlation analyses. Notably, CDKN2A was negatively correlated with FVC% predicted (Fig. S4B, Supplemental Digital Content, http://links.lww.com/MD/L221). CDKN2A and NEDD4 did not significantly correlate with FVC% predicted or DLCO% predicted (Fig. S4C–E, Supplemental Digital Content, http://links.lww.com/MD/L221).

### 3.6. Validation of diagnostic biomarkers

**Figure 8.**
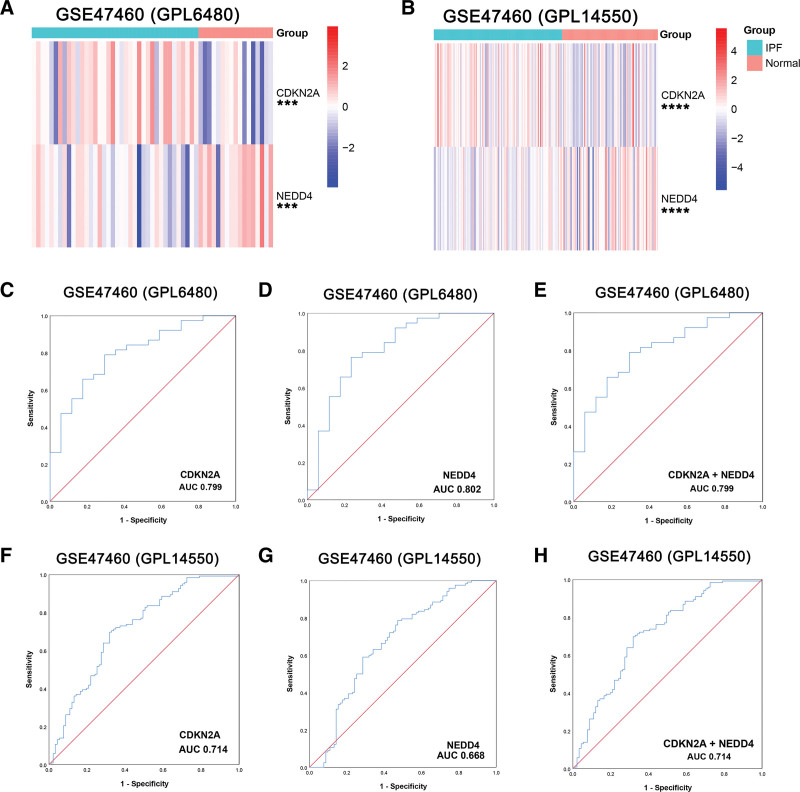
Validation of diagnostic biomarkers in GSE47460 (GPL6480) and GSE47460 (GPF14550). (A) Heatmap of CDKN2A and NEDD4 in GSE47460 (GPL6480). (B) Heatmap of CDKN2A and NEDD4 in GSE47460 (GPL14550). (C-E) ROC analyses results of CDKN2A, NEDD4, and CDKN2A + NEDD4 for IPF in GSE47460 (GPL6480). (F-H) ROC analyses results of CDKN2A, NEDD4, and CDKN2A + NEDD4 for IPF in GSE47460 (GPL14550). ***P < .001, ****P < .0001.

To verify the diagnostic value of CDKN2A and NEDD4, GSE47460 (GPL6480) and GSE47460 (GPL14550) were selected for analysis. In GSE47460 (GPL6480) and GSE47460 (GPL14550), CDKN2A and NEDD4 showed that the difference between IPF and normal controls was statistically significant (Fig. 8A and B). This result was consistent with the derivation group. In GSE47460 (GPL6480), the ROC curves demonstrated that CDKN2A, NEDD4, and CDKN2A + NEDD4 were effective in distinguishing IPF from normal individuals, with AUC values of 0.799, 0.802, and 0.799 (Fig. 8C–E). The AUC of CDKN2A, NEDD4, and CDKN2A + NEDD4 in GSE47460 (GPL14550) was 0.714, 0.668, and 0.714 (Fig. 8F–H). Therefore, CDKN2A and NEDD4 were considered by us as valid diagnostic biomarkers for IPF.

## 4. Discussion

Copper, a metallic trace element, whose homeostasis is essential for various metabolic processes and organ function. Several studies have shown that unbalanced copper homeostasis affects oxidative stress, cytotoxicity, and angiogenesis, among others.^[22,23]^ Cuproptosis is receiving more and more attention as a new type of cell death. Researchers have indicated that copper induces cell death by affecting lipid acylation of the tricarboxylic acid cycle proteins.^[6]^ Meanwhile, it was identified 7 genes conferring resistance to cuproptosis (DLD, DLAT, FDX1, LIAS, LIPT1, PDHA1, and PDHB) and 3 genes facilitating cuproptosis (CDKN2A, GLS, and MTF1).^[6]^ Based on the available studies, it is known that oxidative stress, cytotoxicity and angiogenesis are closely related to IPF.^[24–26]^ However, cuproptosis has not been studied in IPF. Therefore, we aimed to explore effective diagnostic biomarkers of IPF and the role of cuproptosis in its pathogenesis.

In the present study, we initially evaluated the expression levels of CRGs in IPF and normal controls and found statistically significant differences in the expression levels of several CRGs in the 2 groups. Subsequently, IPF patients were divided into 2 clusters based on cuproptosis-related DEGs in order to explore biomarkers for the diagnosis and clustering of IPF. Based on correlation analysis, GO enrichment analysis, ssGSEA, CIBERSORT, ROC and other analyses, the functions of the relevant genes and their predictive ability were determined. Ultimately, we believe that CDKN2A and NEDD4 are excellent predictors for the diagnosis and clustering of IPF.

CDKN2A (p16, INK4A), a tumor suppressor gene, is one of the INK4 class of cell cycle inhibitors,^[27]^ which is involved in the regulation of cell proliferation and apoptosis.^[28]^ CDKN2A is not only one of the CRGs, but also a member of the ferroptosis- and cellular senescence-related genes.^[7,29]^ Consistent with the results of previous studies,^[30,31]^ CDKN2A expression level was upregulated in lung tissue of IPF patients. A previous study has shown that IPF is associated with increased expression level of CDKN2A and induction of cell cycle arrest and cellular senescence.^[32,33]^ And, it was found that CDKN2A was mainly located at the edge of fibroblasts exhibiting epithelial-mesenchymal transition (EMT).^[34]^ Contrary to our results the expression level of CDKN2A was decreased in the peripheral blood of IPF patients and the fibrotic lung tissue of mice.^[35,36]^ We believe that this may be the result of diverse species and tissue samples or differences in gene expression at specific stages of the disease. CDKN2A is a negative regulator of BCR stimulation-induced cell cycle.^[37]^ Senescence, a state of irreversible cell cycle arrest, may be caused by mitochondrial dysfunction.^[38,39]^ Mitochondrial dysfunction has been found to play an important role in the pathogenesis of IPF and fibrosis in animal models.^[40,41]^ The pathological mechanism of cuproptosis includes disruption of mitochondrial metabolic enzymes.^[42]^ Therefore, we conclude that CDKN2A may influence pulmonary fibrosis and molecular subtypes by affecting mitochondrial function.

NEDD4, a member of the HECT E3 ubiquitin ligase family, is divided into 2 subtypes: NEDD4-1 (also called NEDD4) and NEDD4-2 (also called NEDD4L).^[43,44]^ NEDD4, one of the intersecting genes of cuproptosis- and ferroptosis-related genes, is found to be expressed in lung epithelial cells.^[45]^ NEDD4 has been shown to promote the endocytosis of fibroblast growth factor receptor 1 (FGFR1) and downregulate signaling pathway transduction.^[46]^ It was reported that FGFR1 expression level was increased in the lung tissue of IPF patients.^[47]^ In our study, NEDD4 was downregulated in the lung tissue of IPF patients, which is consistent with the results of previous studies mentioned above. It was shown that knockdown of NEDD4 in HEK293T cells enhanced the level of smad1 and the stimulation of transforming growth factor-β1.^[48]^ Surprisingly, induction of EMT by M2 macrophages through the TGF-β/Smad2 signaling pathway was demonstrated in a mouse model of bleomycin-induced pulmonary fibrosis.^[49]^ Overexpression of the TGF-β-related signaling factor Smad3 was also observed in lung tissues of IPF patients.^[50]^ However, NEDD4 enhances TGF-β/smad/EMT signal transduction by directly binding to TGF-β type I receptor to mediate tumor progression.^[51]^ This different conclusion may be due to the diverse mechanisms of gene action in varied diseases. Therefore, we believe that the downregulation of NEDD4 may enhance TGF-β stimulation and EMT to promote pulmonary fibrosis. NEDD4L deficiency in lung epithelial cells leads to progressive lung fibrosis in mice.^[52,53]^ However, the relationship between NEDD4 and IPF is still unclear and needs to be studied extensively. This study is the first to be conducted on NEDD4 in IPF.

However, there are still some limitations in this study. First, we performed the analysis using public datasets without experimental validation. Second, the regulatory mechanisms of CDKN2A and NEDD4 on IPF have not been investigated. Third, the association of CDKN2A and NEDD4 with disease severity could not be determined due to the lack of information on clinical characteristics. Finally, we lack survival information to determine the prognostic value of CDKN2A and NEDD4.

## 5. Conclusion

In conclusion, our study revealed that CDKN2A and NEDD4 were associated with immune cell infiltration and elucidated significant immune heterogeneity among patients with cuproptosis-related molecular clusters of IPF. CDKN2A and NEDD4 were selected as valid diagnostic biomarkers for IPF and even for the assessment of IPF cuproptosis-related molecular clusters. Our study first determined the role of cuprotosis in IPF and 2 identified biomarkers that were useful for IPF diagnosis and copper-related clustering.

## Author contributions

**Conceptualization:** Qi Wang, Yu Shang, Shangwei Ning, Hong Chen.

**Data curation:** Qi Wang, Yu Shang.

**Formal analysis:** Qi Wang, Yu Shang.

**Funding acquisition:** Hong Chen.

**Methodology:** Qi Wang, Yupeng Li, Xincheng Li, Xue Wang.

**Supervision:** Yupeng Li, Shangwei Ning.

**Validation:** Yu Shang, Yaowu He, Jing Ma.

**Visualization:** Qi Wang, Yaowu He.

**Writing – original draft:** Qi Wang.

**Writing – review & editing:** Qi Wang, Yu Shang, Shangwei Ning, Hong Chen.

## Supplementary Material
