# Supplementary figures and images for: Identification of cuproptosis-related diagnostic biomarkers in idiopathic pulmonary fibrosis
Source: Medicine (Baltimore). 2024 Jan 12;103(2):e36801. doi: 10.1097/MD.0000000000036801 (PMC10783416; doi:10.1097/MD.0000000000036801)

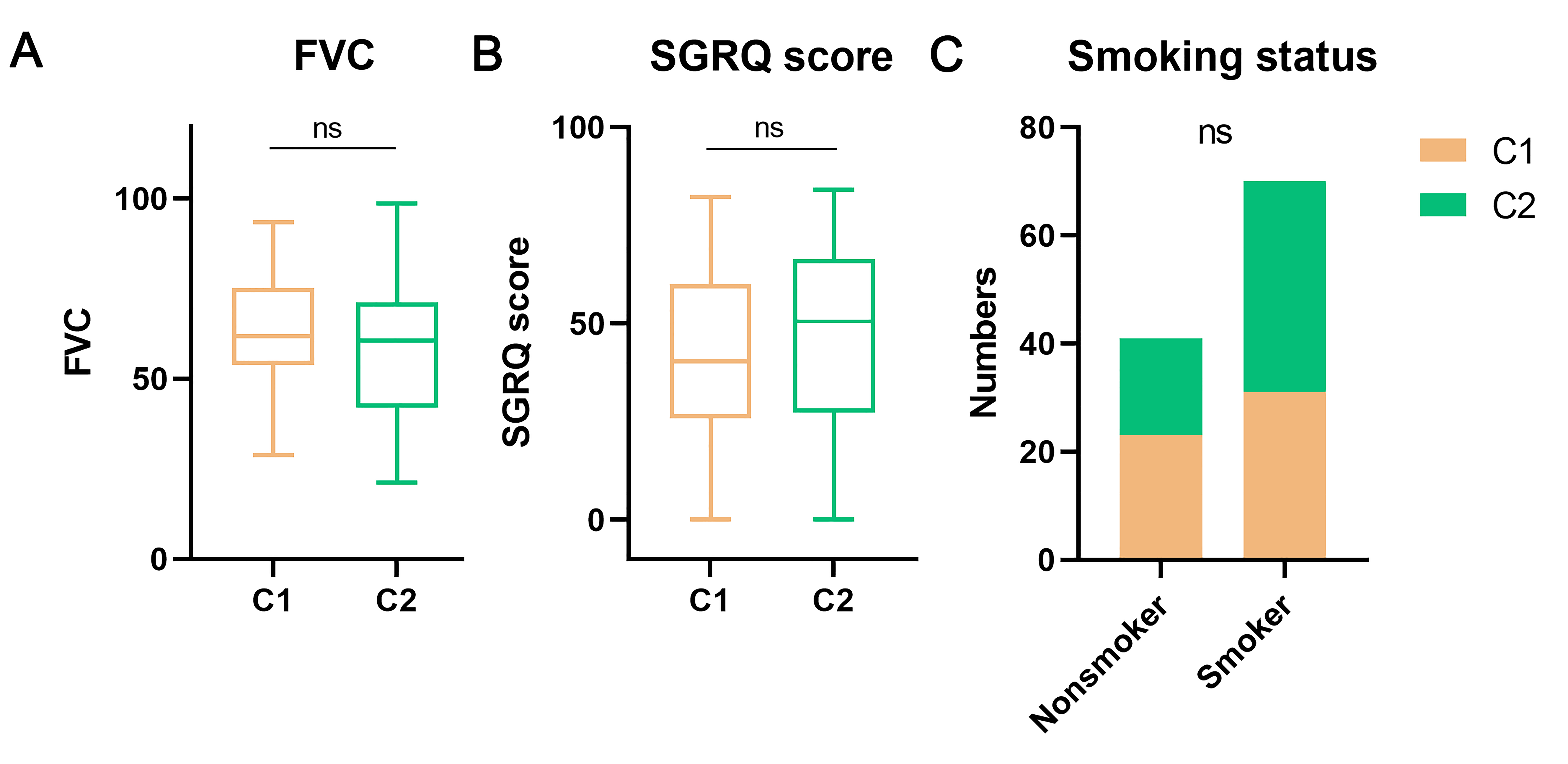

Supplement: Supplementary file 4 [file medi-103-e36801-s004.tif]

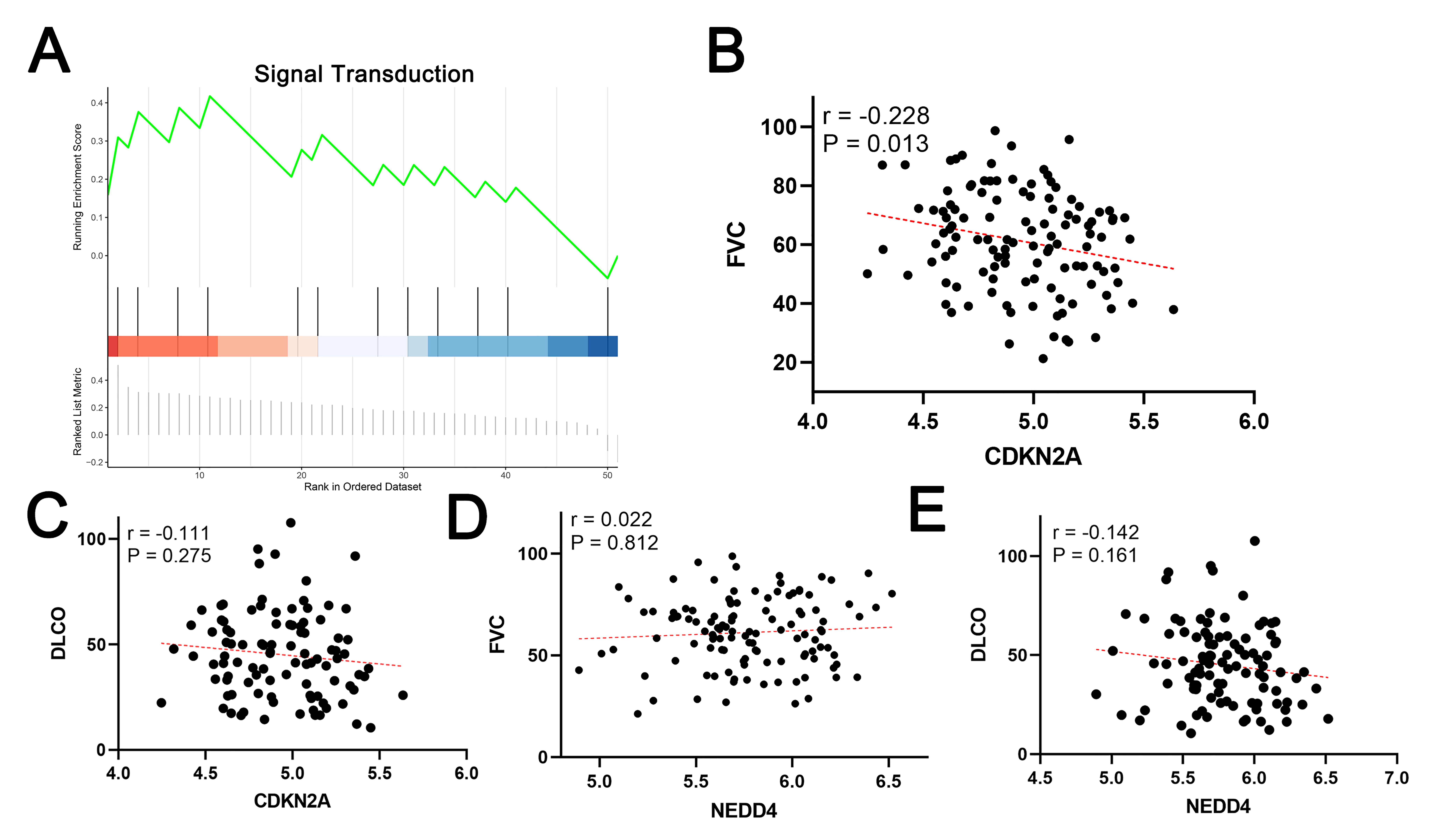

Supplement: Supplementary file 7 [file medi-103-e36801-s007.tif]
